# Quercetin Exposure Suppresses the Inflammatory Pathway in Intestinal Organoids from Winnie Mice

**DOI:** 10.3390/ijms20225771

**Published:** 2019-11-16

**Authors:** Manuela Dicarlo, Gabriella Teti, Giulio Verna, Marina Liso, Elisabetta Cavalcanti, Annamaria Sila, Sathuwarman Raveenthiraraj, Mauro Mastronardi, Angelo Santino, Grazia Serino, Antonio Lippolis, Anastasia Sobolewski, Mirella Falconi, Marcello Chieppa

**Affiliations:** 1 National Institute of Gastroenterology “S. de Bellis”, Institute of Research, 70013 Castellana Grotte (BA), Italy; giu.verna@gmail.com (G.V.); marinaliso@libero.it (M.L.); elisabetta.cavalcanti@irccsdebellis.it (E.C.); a.sila@outlook.it (A.S.); mauro.mastronardi@irccsdebellis.it (M.M.); grazia.serino@irccsdebellis.it (G.S.); antonio.lippolis@irccsdebellis.it (A.L.); 2 Department of Biomedical and Neuromotor Sciences-DBNS, Università di Bologna, Via Irnerio 48, 40126 Bologna, Italy; gabriella.teti2@unibo.it (G.T.); mirella.falconi@unibo.it (M.F.); 3 School of Pharmacy, University of East Anglia, Norwich Research Park, Norwich NR4 7TJ, UK; S.Raveenthiraraj@uea.ac.uk (S.R.); A.Sobolewski@uea.ac.uk (A.S.); 4 ISPA-CNR, Institute of Science of Food Production, C.N.R. Unit of Lecce, 73100 Lecce, Italy; angelo.santino@ispa.cnr.it; 5 Department of Immunology and Cell Biology, European Biomedical Research Institute of Salerno (EBRIS), 84125 Salerno, Italy

**Keywords:** inflammatory bowel diseases, quercetin, small intestinal organoids

## Abstract

Inflammatory bowel diseases (IBDs) are chronic and relapsing immune disorders that result, or possibly originate, from epithelial barrier defects. Intestinal organoids are a new reliable tool to investigate epithelial response in models of chronic inflammation. We produced organoids from the ulcerative colitis murine model Winnie to explore if the chronic inflammatory features observed in the parental intestine were preserved by the organoids. Furthermore, we investigated if quercetin administration to in vitro cultured organoids could suppress LPS-induced inflammation in wild-type organoids (WT-organoids) and spontaneous inflammation in ulcerative colitis organoids (UC-organoids). Our data demonstrate that small intestinal organoids obtained from Winnie mice retain the chronic intestinal inflammatory features characteristic of the parental tissue. Quercetin administration was able to suppress inflammation both in UC-organoids and in LPS-treated WT-organoids. Altogether, our data demonstrate that UC-organoids are a reliable experimental system for investigating chronic intestinal inflammation and pharmacological responses.

## 1. Introduction

Inflammatory bowel diseases (IBDs) are chronic and relapsing immune disorders that include Crohn’s disease (CD) and ulcerative colitis (UC), which can be discriminated based on the site of inflammation and the histological alterations in the gut wall. Although the exact IBD etiology is still unknown, a combination of multiple host (genetic predisposition and cellular and immune changes) and environmental (e.g., diet, smoking, and chronic stress) factors has been proposed [1]. Increasing evidence demonstrated that dysfunctions in the intestinal epithelium, a monolayer of cells that lies on the lamina propria, play a crucial role in IBD pathogenesis [2]. 

The most common experimental approaches to investigate IBD pathogenesis and/or to evaluate their therapeutic options have been animal models and traditional two-dimensional (2-D) in vitro cell cultures, using cell lines. The ethical issues of performing in vivo animal experiments, as well as the less biologically relevant use of immortalized or transformed cell lines, paved the way for the development of alternative experimental systems [3].

Gut organoids are three-dimensional (3-D) in vitro models of the gut epithelium that can be isolated and grown from adult mucosal tissues (intestinal crypts) or by differentiation of embryonic (ESCs) or induced pluripotent stem cells (iPSC). These “mini-intestines” display a typical polarized intestinal epithelium subdivided in villus-like zones and crypt-like domains (“buds”). Indeed, in vitro intestinal organoids are composed of similar differentiated epithelial phenotypes and relative proportions that are present in the parental gut [4]. Interestingly, mini-intestines recapitulating the complex architecture of in vivo gut epithelium have offered the opportunity to study physiological conditions (intestinal development, nutrient and/or drug absorption, etc.) and pathological disorders, including IBDs and cystic fibrosis [3,5,6].

Our laboratory obtained a spontaneous, progressive model of UC (Winnie). These mice are characterized by a point mutation in the Muc2 gene that results in an unresolved endoplasmic reticulum stress and consequent chronic intestinal inflammation [7]. The Winnie model could be particularly relevant for exploring UC prevention strategies due to the slow progression toward the UC phenotype. 

We recently demonstrated the potential for using nutritionally derived polyphenols as an adjuvant for IBD treatment through regulation of immune cells and their secretion of inflammatory mediators [8]. Quercetin is a plant-derived polyphenolic compound belonging to the flavonols (a subclass of flavonoids) and has shown beneficial effects in the prevention and/or treatment of several pathological conditions, due to its anti-inflammatory, antioxidant, and antitumoral activities [9,10]. Our previous studies showed that quercetin reduced the secretion of several inflammatory cytokines, including TNF-α, from murine bone-marrow-derived dendritic cells (BMDCs) [11]. We also demonstrated that the molecular mechanism was secretory leukoprotease inhibitor (Slpi)-dependent and involved extracellular iron transport [12,13,14].

In this study, we used 3-D intestinal organoids obtained from wild-type (WT) or Winnie mice (UC-organoids) to determine intestinal epithelial responses in a quercetin-enriched environment. Organoids obtained from WT and Winnie mice respond to quercetin by suppressing the expression of TNF-α, CCAAT/enhancer binding protein β (C/EBP-β), and lipocalin-2 (Lcn-2) and upregulating Slpi, heme oxygenase 1 (Hmox1), and ferroportin 1 (Fpn1). Remarkably, we detected proliferative, morphological, and molecular differences between our organoid populations. Indeed, UC-organoids frequently showed a greater growth rate during subculture and displayed less organized structures, together with an inflammatory profile. Overall, our findings suggest that the intestinal epithelium responds to quercetin exposure by suppressing the inflammatory response. Furthermore, UC-organoids representing a reliable tool that better resembles IBD inflammatory epithelium could be used for various purposes (dietary investigations, therapeutic applications, etc.). 

## 2. Results

### 2.1. Growth and Ultrastructural Comparison between WT- and UC-Organoids

**Figure 1:**
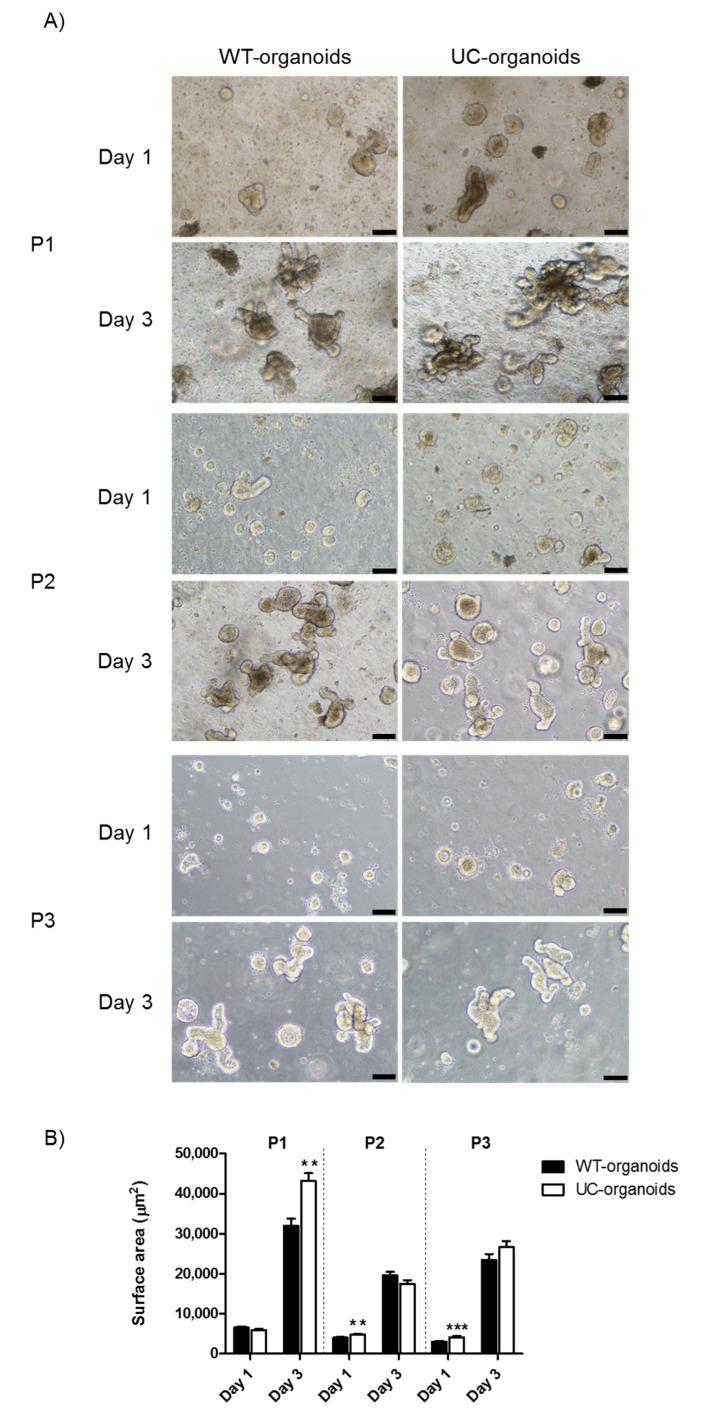
Proliferation of WT- and UC-organoids over time. (**A**) Representative images of WT- and UC-organoids on day one and three, after seeding in Matrigel. Magnification 10×, scale bars 100 μm. (**B**) Average surface area of organoids. Histograms represent mean surface area (μm^2^) ± SEM of four independent experiments. Unpaired two-tailed Student’s t-test was used for statistical analysis. *** *p* < 0.001, ** *p* < 0.01.

In order to evaluate potential growth differences between WT- and UC-organoids, we measured their surface area on day one and three for the initial three subpassages (P1–P3). On day one, both WT- and UC-organoids were prevalently characterized by a rounded morphology. However, UC-organoids were larger in respect to WT ones at P2 (4812 ± 184 vs. 3986 ± 198) and P3 (4140 ± 234 vs. 3018 ± 192) (** *p* < 0.01 and *** *p* < 0.001, respectively). On day three, WT- and UC-organoids displayed a mature morphology, but UC-organoids were significantly bigger than WT controls at P1 (43,269 ± 1879 vs. 31,974 ± 1732) (*p* < 0.01) (Figure 1A,B). 

**Figure 2:**
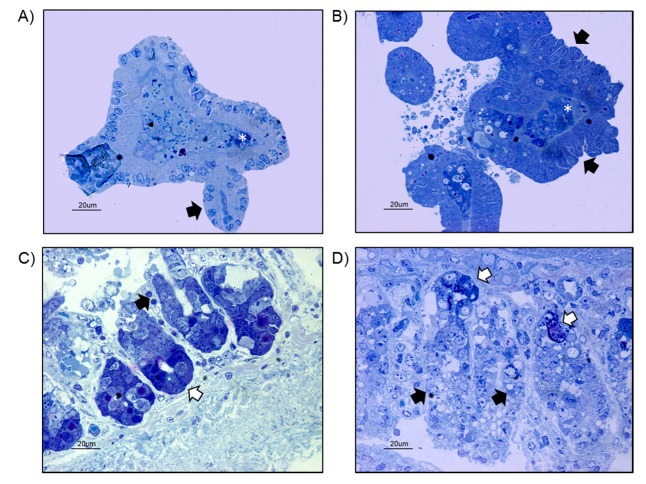
(**A**) Light microscopy analysis of blue toluidine stained WT-organoids showing enterocytes arranged in a round irregular monolayer and delimiting a central lumen in which some necrotic cells were observed (white asterisk). Some buds, mimicking the intestinal crypts, extended from the organoid (black arrow) (bar: 20 μm). (**B**) Light microscopy analysis of UC-organoids, showing enterocytes arranged in an irregular multilayer structure (black arrows), surrounding a necrotic area (white asterisk) (bar: 20 μm). (**C**) Small intestine from WT mice, showing intestinal villi in longitudinal section. Enterocytes (black arrow) and Paneth cells (white arrow) were observed (bar: 20 μm). (**D**) Small intestine from Winnie mice showing intestinal villi in longitudinal section. A less-organized morphology was observed in which enterocytes (black arrow) and Paneth cells (white arrow) were hardly recognizable (bar: 20 μm).

In order to evaluate whether gut organoids obtained from Winnie mice resembled WT-organoids, we compared the morphology and the ultrastructural organization of WT- and UC-organoids. Light microscopy analysis showed WT-organoids displayed a characteristic rounded morphology, with some buds or crypt domains extending outward and necrotic cells located in the central lumen (Figure 2A). Enterocytes, characterized by the presence of the nucleus in the basal region, delimited the organoid structure. Organoids grown from the small-intestinal crypts isolated from Winnie mice exhibited an irregular morphology (Figure 2B). Enterocytes showed a more disorganized arrangement and delimited a central lumen characterized by the presence of some necrotic cells (Figure 2B). In order to better clarify the morphology of small-intestinal organoids, a comparison with small-intestine biopsies isolated from WT and Winnie mice was performed. The WT small intestine showed the presence of several intestinal villi in which enterocytes and Paneth cells were observed (Figure 2C). A less-organized arrangement of small-intestinal villi was observed in Winnie mice, in which enterocytes and Paneth cells were hardly distinguished (Figure 2D). 

**Figure 3:**
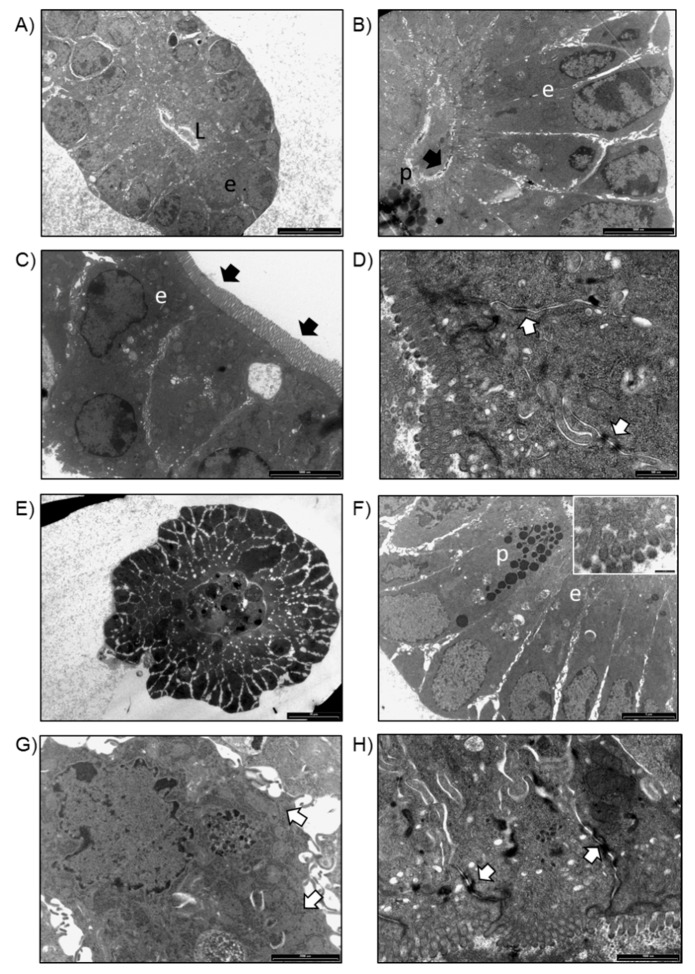
(**A**) TEM image of organoids obtained from WT intestinal crypts. Enterocytes (e) were organized in a round structure delimiting a central lumen (L), resembling an intestinal villus in transversal section (bar: 10 μm). (**B**) Enterocytes (e) showing a polarized organization, with nuclei located close to the basal side of the cell and several microvilli at the apical surface (black arrow). Paneth cells (p) were observed (bar: 500 nm). (**C**) Detail of microvilli on the apical surface of the enterocytes (e) (bar: 2000 nm). (**D**) Desmosomes on the lateral surface of enterocytes were detected (white arrows) (bar: 500 nm). (**E**) TEM analysis of organoids obtained from Winnie intestinal crypts (bar: 20 μm). (**F**) Paneth cells (p), characterized by the presence of several granules and enterocytes (e), were observed (bar: 5 μm). On the apical surface of enterocytes, short microvilli were detected (insert; bar: 200 nm). (**G**) Detail of an enterocyte, showing a dilated RER (white arrows) (bar: 200 nm). (**H**) Desmosomes on the lateral surface of enterocytes were detected (bar: 500 nm).

TEM analysis of WT-organoids showed the presence of enterocytes and Paneth cells organized in round-shaped structures resembling intestinal villi in a transversal section (Figure 3A). Enterocytes showed a polarized organization with basally located nuclei (Figure 3B). At higher magnifications, several small microvilli extruding from the apical membrane of the cells were distinguished (Figure 3C), and desmosomes on the lateral surfaces of enterocytes were observed (Figure 3D). Organoids obtained from Winnie intestines showed a round-shaped organization in which enterocytes and Paneth cells were observed (Figure 3E). At higher magnification, Paneth cells showed several cytoplasmic granules (Figure 3F), while enterocytes showed some cytoplasmic vacuoles and dilated rough endoplasmic reticulum (RER) (Figure 3F,G). A reduced amount and shorter microvilli were observed on the apical surface of the cells (Insert in Figure 3F). Compared to organoids obtained from WT, enterocytes of UC-organoids were loosely connected to each other, as frequent white areas were observed by TEM (Figure 3E). Despite this, no difference in desmosomes was revealed between WT- and UC-organoids (Figure 3D,H). 

**Figure 4:**
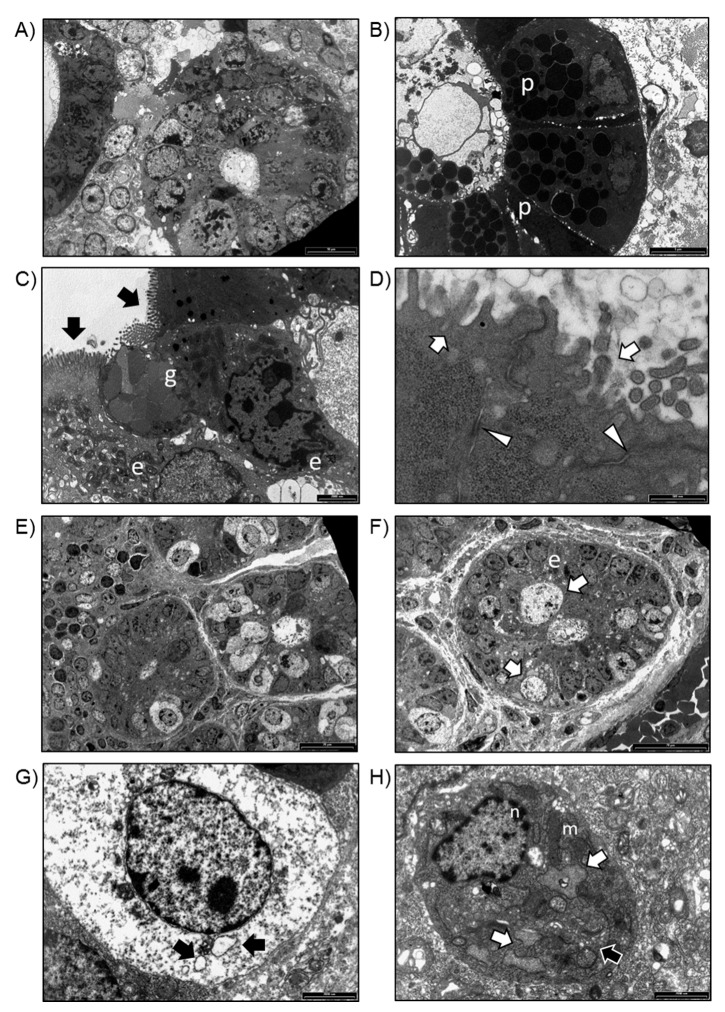
(**A**) TEM analysis of intestinal biopsies isolated from WT mice. Villi in transversal sections (bar: 20 μm). (**B**) Transversal section of an intestinal villus. Paneth cells (p) were observed (bar: 20 μm). (**C**) Goblet cells (g) and enterocytes (e) with basally located nuclei and microvilli (black arrows) on the apical surface of the cells were observed (bar: white arrows) (bar: 500 nm). (**D**) Detail of microvilli on the apical surface of enterocytes (white arrows) and desmosomes on the lateral surface of enterocytes (arrowheads) (bar: 500 nm). (**E**) TEM analysis of intestinal biopsies isolated from Winnie mice. Transversal section of intestinal villi (bar: 20 μm). (**F**) Detail of an intestinal villus in which enterocytes (e) and distended cells were observed (bar: 20 μm). (**G**) Distended cell containing several vacuoles (black arrows) (bar: 2000 nm). (**H**) Enterocyte showing a preserved nucleus (n), dilated RER (white arrows), several mitochondria (m), and enlarged Golgi complex (black arrow) (bar: 2000 nm).

To determine whether in vitro gut organoids resemble the in vivo intestinal epithelium in WT and Winnie mice, we next compared their ultrastructure. The intestinal wall of WT mice showed several villi in transversal sections (Figure 4A) in which enterocytes, Paneth cells, and goblet cells were distinguished (Figure 4B,C). Enterocytes showed several microvilli on their apical surface (Figure 4C) and desmosome junctions on the lateral surfaces (Figure 4D); moreover, well-preserved organelles, such as mitochondria, RER, and Golgi complex, were observed. TEM analysis of Winnie intestinal biopsies showed several villi in transversal sections, in which enterocytes and distended cells with vacuolar material were visible (Figure 4E,F). At higher magnification, distended cells showed abundant cytoplasmic vacuoles (Figure 4G), whilst enterocytes were characterized by the presence of several dilated RER, enlarged Golgi complex (Figure 4H), and mitochondria with enlarged cristae (Figure 4H). 

### 2.2. Comparison of Endogenous Gene Expression of WT- and UC-Organoids 

**Figure 5:**
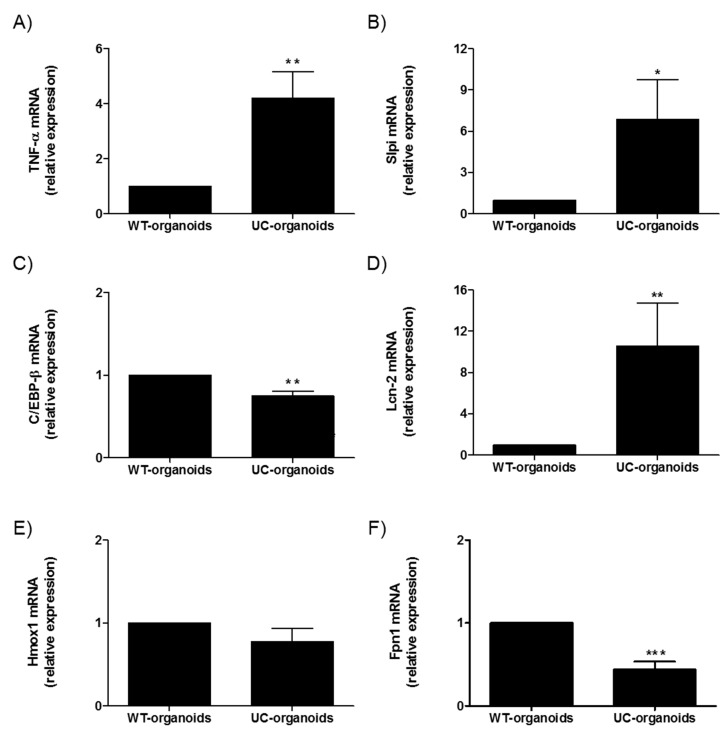
Comparison between small-intestinal WT- and UC-organoids. Expression of TNF-α (**A**), Slpi (**B**), C/EBP-β (**C**), LCN-2 (**D**), Hmox (**E**), and Fpn1 (**F**) was detected by real-time PCR. Relative expression of selected genes was calculated relative to WT-organoids. Histograms represent mean expression ± SEM of four independent experiments. Unpaired two-tailed Student’s t-test was used for statistical analysis. *** *p* < 0.001, ** *p* < 0.01, * *p* < 0.05.

We next compared the molecular expression of selected genes in WT- and UC-organoids. A significant upregulation of TNF-α was detected in UC-organoids, which was in line with what was observed in the parental tissue (** *p* < 0.01). Similarly, Slpi and Lcn-2 were upregulated (* *p* < 0.05 and ** *p* < 0.01, respectively), while C/EBP-β (** *p* < 0.01), Hmox1, and Fpn1 (*** *p* < 0.001) were downregulated (Figure 5).

### 2.3. Effect of Quercetin on WT Intestinal Organoids 

**Figure 6:**
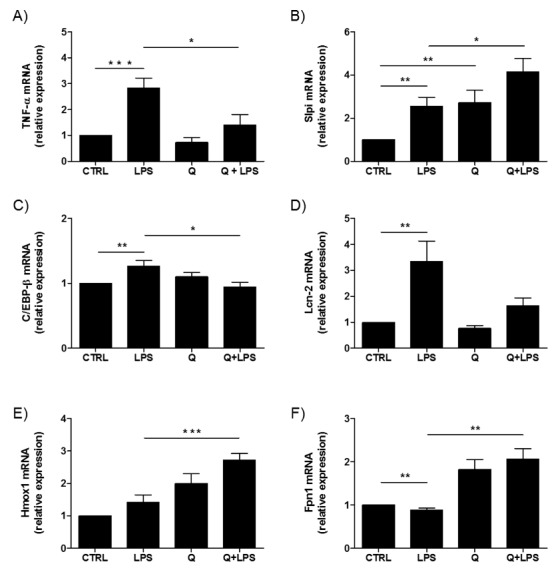
The effect of quercetin on WT intestinal organoids. Expression of TNF-α (**A**), Slpi (**B**), C/EBP-β (**C**), LCN-2 (**D**), Hmox (**E**), and Fpn1 (**F**) was detected by real-time PCR. Relative expression of selected genes was calculated relative to untreated WT-organoids (control). Histograms represent mean expression ± SEM of four independent experiments. Unpaired two-tailed Student’s t-test was used for statistical analysis. *** *p* < 0.001, ** *p* < 0.01, * *p* < 0.05.

To evaluate the organoid response to the inflammatory stimulus (LPS), organoids from WT small intestines were cultured in the presence of 1µM LPS and the mRNA collected 6 h later. We used the same quercetin concentration of our previous studies focused on DCs [11,12,13,14]. TNF-α expression was significantly increased in LPS-treated organoids compared to control (*** *p* < 0.001). Quercetin treatment significantly reduced the LPS-induced TNF-α expression (* *p* < 0.05) (Figure 6A).

Due to the crucial role of Slpi expression in suppressing LPS-induced inflammation in BMDCs [12,13], we investigated whether quercetin affected Slpi induction. Quercetin exposure promoted Slpi expression (*p* < 0.01) in WT-organoids. Organoids treated with LPS also upregulated Slpi (** *p* < 0.01), but the induction was significantly higher if the organoids were pretreated with quercetin (* *p* < 0.05) (Figure 6B). A similar pattern of expression was detected for C/EBP-β. Indeed, C/EBP-β mRNA expression was higher in LPS-treated WT-organoids (* *p* < 0.01) compared to control, and with quercetin downregulating this effect (* *p* < 0.05) (Figure 6C).

Lcn-2, which was highly expressed in LPS-treated organoids (** *p* < 0.01), showed a slight increase when WT organoids were exposed to quercetin in the presence of LPS (Figure 6D).

Based on our previous results demonstrating the effects of quercetin on iron homeostasis in BMDCs [14], we assessed the expression of Hmox1 and Fpn1 mRNA. Interestingly, we found a significant increase of Hmox1 and Fpn1 expression in WT-organoids exposed to quercetin in the presence of LPS (*** *p* < 0.001 and ** *p* < 0.01, respectively) (Figure 6E,F). Fpn1 was downregulated in LPS-treated organoids (** *p* < 0.01).

### 2.4. Effect of Quercetin on Winnie Intestinal Organoids

**Figure 7:**
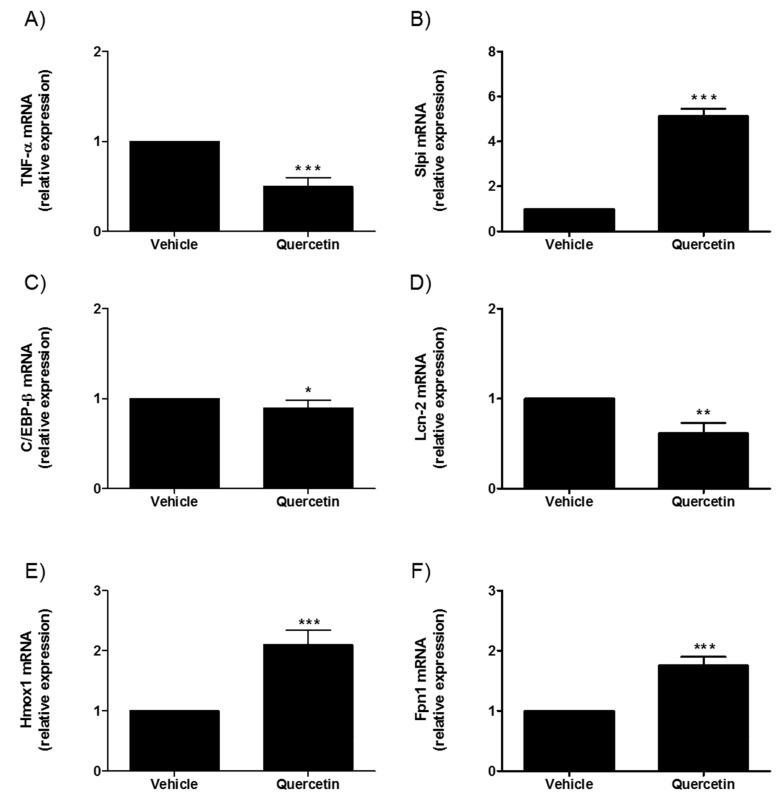
Effect of quercetin on small-intestinal UC-organoids. Expression of TNF-α (**A**), Slpi (**B**), C/EBP-β (**C**), LCN-2 (**D**), Hmox (**E**), and Fpn1 (**F**) was detected by real-time PCR. Relative expression of selected genes was calculated relative to untreated UC-organoids. Histograms represent mean expression ± SEM of four independent experiments. Unpaired two-tailed Student’s t-test was used for statistical analysis. *** *p* < 0.001, ** *p* < 0.01, * *p* < 0.05.

As previously demonstrated with Winnie mice, Slpi, Hmox1, and TNFα intestinal expression is regulated by a quercetin-enriched diet [15]. Quercetin treatment of UC-organoids caused a significant decrease of the inflammatory-related genes TNFα, C/EBP-β and Lcn-2 (*** *p* < 0.001, * *p* < 0.05 and ** *p* < 0.01, respectively), conversely genes associated with inflammatory suppression and tissue repair (Slpi, Hmox1, and Fpn1) were induced by quercetin exposure (*** *p* < 0.001) (Figure 7). Of note, genes related with epithelial permeability (tight junction protein Cldn-1, Cldn-2, Cldn-4, and Ocln) did not change in response to quercetin exposure in UC-organoids.

## 3. Discussion

The gut epithelial cell barrier is a remarkable defense system that acts as a physical, chemical, and electrical barrier [16]. This crucial function relies on several different strategies, including tight junctions that regulate the paracellular permeability, a mucus layer covering the luminal epithelial surface, and antimicrobial peptide secretion, which provides primary protection against microorganisms [17]. Intestinal barrier function depends upon specialized cell types (absorptive enterocytes, goblet cells, enteroendocrine cells, Paneth cells, and tuft cells) that originate from epithelial stem cells located within the base of the intestinal crypts [18]. It is well-known that failure of gut barrier function contributes to IBD pathogenesis and permits the intestinal microbiota to contact the underlying immune cells in the lamina propria, causing an immune response [2,19].

Recent discovery of intestinal adult stem cells and, consequently, the development of procedures to generate intestinal organoids have provided an innovative 3-D model that could replace traditional experimental systems (i.e., 2-D cell lines and in vivo models). Organoids could become the most important experimental system for various applications, including gut-disease modelling, intestinal organogenesis, host–pathogen interactions, regenerative medicine, etc. [3,5,6].

In this study, we treated gut organoids with quercetin to evaluate the contribution of gut epithelial cells to the inflammatory suppression we previously observed following polyphenol oral administration to mice (insert the reference). First, we compared WT gut organoids and organoids from the Winnie mouse, a murine model of spontaneous UC that could better mimic IBD [7]. In vitro Winnie organoids displayed similar morphology to WT the day after seeding, and both exhibited villi and crypt-like domains following several passages. Specialized cell types such as enterocytes and Paneth cells were present in WT- and UC-organoids and distributed as in the in vivo setting. Remarkably, although cell-to-cell interactions, such as desmosomes, were preserved in WT- and Winnie organoids, the UC-organoids appeared less organized than WT mini-guts. Notably, the average area of UC-organoids was larger than WT controls, possibly because of the inflammatory microenvironmental milieu. Following inflammatory LPS-stimulation, gut organoids upregulated inflammatory mediators, such as TNF-α and LCN-2. LCN-2 is also known as neutrophil gelatinase-associated lipocalin (NGAL) and is highly expressed by intestinal epithelial cells in inflammatory disorders, including in IBD [20]. The anti-inflammatory activity of LCN-2 is due to its capacity to sequestrate iron, limiting the bacterial growth and blocking gut dysbiosis during intestinal inflammation [20]. LCN-2 expression is also linked to TNF-α production; indeed, a previous study on human colonic cell lines demonstrated that LCN-2 is regulated by TNF-α and other Th17 cytokines (i.e., IL-17A and IL-22) [21]. 

Strikingly, quercetin suppressed these inflammatory genes induced by LPS, decreasing the expression levels of both TNF-α and LCN-2 mRNA. As previously observed in BMDCs [12,13], we found that quercetin was able to upregulate Slpi expression in WT-organoids. Slpi is a key serine proteinase inhibitor, with potent antimicrobial activity that is mostly produced by immune cell types (i.e., DCs, neutrophils and macrophages) [22]. However, Slpi production by human intestinal epithelium has been demonstrated by Si-Tahar et al. and suggests its protective role against harmful attacks of microbes, digestive enzymes, and inflammatory cells [23]. 

Notably, the anti-inflammatory effect of quercetin was also coupled with a reduced expression of C/EBP-β, a transcription factor that triggers the expression of various inflammatory mediators, including TNF-α [24]. Gut epithelial reduction in C/EBP-β expression and consequent inhibition of the inflammatory response following quercetin administration is consistent with earlier reports on both epithelial cell lines [25,26] and BMDCs [27]. 

We previously observed that the anti-inflammatory ability of quercetin was strictly associated with iron-sequestration in the extracellular milieu. Quercetin supplementation in the BMDCs culture media affected DCs’ iron metabolism, inducing the upregulation of Hmox, a cytosolic enzyme that produced ferrous irons, and Fpn1, which allows iron extracellular export [14]. In line with these data, in this study, we found that quercetin exposure upregulated Hmox and Fpn1 mRNA expression, even in the absence of LPS administration. Our results are in contrast with Lesjak M. et al., who reported a significant decrease in Fpn1 mRNA expression in the intestinal cell line (Caco2) exposed to quercetin [28], but this discrepancy may be due to a different experimental protocol and the use of primary epithelial cells versus a Caco2 cell line. 

Interestingly, UC-organoids shared a similar inflammatory profile to that of LPS-treated WT-organoids, as revealed by the upregulation of TNF-α, LCN-2 and Slpi mRNA. It was expected that LCN-2 and Slpi expression would be higher in UC-organoids, as Heazlewood et al., who first described the Winnie model, found their increased expression in the intestinal tissues of these mice [7]. Furthermore, we have already demonstrated that inflamed colonic tissues expressed Slpi as a likely anti-inflammatory protective mechanism [11]. As far as the iron metabolism is concerned, we found a molecular pattern similar to that of WT mini-gut LPS-stimulated with a superimposable Hmox expression level and the downregulation of Fpn1. Surprisingly, C/EBP-β mRNA expression was downregulated in UC-organoids. Nonetheless, C/EBP-β expression level decreased in quercetin-treated UC-organoids. In line with our previous study on Winnie mice fed with a polyphenol-enriched diet [15], here we found that the presence of quercetin was able to dampen the inflammatory profile of UC-organoids, as demonstrated by the downregulation of both TNF-α and LCN-2 mRNA and the induction of inflammatory suppressors (i.e., Slpi, Hmox1, and Fpn1). Taken together, these data show that the Winnie organoid model mirrors the inflammatory status of the epithelium during inflammation, similar to that of LPS, and that quercetin can modulate inflammatory responses in both settings.

Intestinal organoids have already been used as 3-D models for nutritional studies, in order to evaluate the effects of various dietary nutrients and polyphenolic compounds on the intestinal growth and development [29,30]. Currently, the impact of quercetin on intestinal epithelium functions has been tested in vitro, using human colonic epithelial cell line Caco-2 [31,32,33] and self-renewing monolayers of primary colonic or rectal epithelial cells [34]. Hence, to the best of our knowledge, this is the first study that used 3-D gut organoids to demonstrate the anti-inflammatory effects of quercetin on intestinal epithelial cells and, consequently, its beneficial role on gut health. We recognize that results could become more relevant when confirmed by using human organoids from UC patients and healthy controls; nonetheless, the present study represents an important experimental setup. Future studies will be able to take advantage of the results described in the present study, in order to evaluate the efficiency of new pharmacological interventions for chronic inflammatory syndromes of the gastrointestinal tract. 

## 4. Materials and Methods 

### 4.1. Ethics Statement

Our experiments were conducted in agreement with national and international guidelines and were approved by the authors’ institutional review board (Organism for Animal Well-Being—OPBA). All animal experiments were carried out in accordance with Directive 86/609 EEC, enforced by Italian D.L. n. 116/1992, and approved by the Committee on the Ethics of Animal Experiments of Ministero della Salute–Direzione Generale Sanità Animale (Prot. 768/2015-PR 27/07/2015) and the official RBM veterinarian. Animals were sacrificed if found to be in a severe clinical condition, in order to avoid undue suffering.

### 4.2. Isolation of Mouse Small-Intestinal Crypts and Organoid Culture

Intestinal crypts were isolated from the small intestine of 4 wild-type mice purchased from Jackson Laboratories (C57BL/6, Stock No.: 000664) or from 4 Winnie mice obtained from the University of Tasmania, according to the protocol suggested by Stemcell Technologies Inc. After harvesting, the small intestine was flushed with cold Dulbecco’s phosphate buffered saline (DPBS) (Gibco, Waltham, MA, USA), in a petri dish, and cut longitudinally. The resulting intestinal sheet was washed several times with cold DPBS and then minced into 2 mm fragments. After being transferred into a 50 mL conical tube, tissue pieces were washed several times until the supernatant was clear. Next, intestinal fragments were incubated with Gentle Cell Dissociation Reagent (GCDR) (Stemcell Technologies Inc., Vancouver, Canada), at room temperature, for 15 min, on a rocking platform. To detach the intestinal crypts from the basal membrane, GCDR was replaced with cold DPBS + 0.1% bovine serum albumin (BSA) (Sigma-Aldrich, St. Louis, MO, USA), and a mechanical force (pipetting) was imposed. Crypt-enriched supernatants were filtered through a 70 μm cell strainer (Corning, NY, USA) in a new 50 mL conical tube. The pipetting/filtration procedure was repeated four times, and then the fractions containing abundant amounts of intestinal crypts were selected for culturing and centrifuged at 200× *g*, for 5 min, to eliminate single-cell contamination. 

After counting with an inverted microscope, the isolated intestinal crypts were re-suspended in room-temperature IntestiCult™ Organoid Growth Medium (Stemcell Technologies Inc., Vancouver, Canada) and mixed 1:1 with ice-cold Matrigel Matrix (Corning, NY, USA). For organoid generation, intestinal crypts were plated in 24-well tissue culture plates (Corning, NY, USA) (200 crypts per 50 μL per well) and maintained at 37 °C, for 10 min, to allow Matrigel domes to polymerize. Next, 750 μL of room-temperature IntestiCult™ Organoid Growth Medium was added to each well, and the plates were further incubated at 37 °C and 5% CO_2_. The culture media were changed three times per week. Intestinal organoids were split when they showed dark necrotic cores and used for experiments, starting from the third passage.

### 4.3. Organoid Growth Assessment

To evaluate WT- and UC-organoid growth during their subculture, intestinal crypts were seeded in 24-well tissue culture plates (100 crypts per 50 μL per well) and cultured for 3 days, as described above. Representative images of intestinal organoids were acquired at 1 and 3 days after seeding, using the inverted microscope Eclipse Ti2 (Nikon Instruments, Amsterdam, Netherlands), and the surface area of organoids (μm^2^) was measured in the horizontal direction, with ImageJ Software (National Institute of Health, Bethesda, MD, USA) [35].

### 4.4. TEM Analysis

Intestinal organoids, obtained as previously described, and fragments of intestinal sheets, isolated from the small intestine of WT and Winnie mice, were briefly washed in DPBS and then immediately fixed in 2.5% glutaraldehyde in 0.1 M cacodylate buffer for 24 h, at 4 °C. After several washes in 0.15 M cacodylate buffer, all the samples were post-fixed in 1% OsO4, in cacodylate buffer 0.1 M, for 1 h, at room temperature, gradually dehydrated by graded acetone serial steps and embedded in epoxy resin. Ultrathin slices of 100 nm were stained by uranyl acetate solution and lead citrate, and then observed with transmission electron microscope CM10 Philips (FEI Company, Eindhoven, The Netherlands), at an accelerating voltage of 80 kV. Images were recorded by a Megaview III digital camera (FEI Company, Eindhoven, The Netherlands). Sections of 300 nm were stained with 1% toluidine blue solution and were observed by the light microscope Nikon Eclipse E800 (Nikon, Tokyo, Japan).

### 4.5. Small-Intestinal Crypt Stimulation

Intestinal crypts were seeded in 24-well tissue culture plates (Corning, NY, USA) (100 crypts per 50 μL per well) and maintained at 37 °C and 5% CO_2_, until the appearance of mature organoids (4 days after seeding). Organoid maturation was monitored daily with the inverted microscope Eclipse Ti2 (Nikon Instruments, Amsterdam, Netherlands). Intestinal organoids were incubated with 25 μM of quercetin (Sigma-Aldrich, St Louis, MO, USA). After 4 h, intestinal organoids were stimulated with 1 μg/mL of LPS (L6143, Sigma-Aldrich, St Louis, MO, USA) for 6 h. Mature organoids that did not receive quercetin treatment or LPS stimulation were used as controls for comparative analyses. All experiments were performed in triplicate.

### 4.6. RNA Extraction and qPCR Analysis

Total RNA was isolated from small-intestinal organoids by using TRIzol^®^ (Thermo Fisher Scientific, MA, USA) reagent method, according to manufacturer’s instructions. Then, 1 μg of total RNA was retrotranscribed with the iScript cDNA Synthesis kit (Biorad, CA, USA), using random primers for cDNA synthesis. The expression of TNF-α, Hmox1, Fpn1, Slpi, and Gapdh was assessed with TaqMan gene-expression assay (Thermo Fisher Scientific, MA, USA) murine probes: Mm00443258_m1, Mm00516005_m1, Mm01254822_m1, Mm00441530_g1, and Mm99999915_g1, respectively. For C/EBP-β, qRT-PCR reactions were performed by using SYBR Green chemistry with QuantiTect Primer Assay (Qiagen, Hilden, Germany) and SsoAdvanced Universal SYBR Green Supermix (BioRad Laboratories, Hercules, CA, USA). All qPCR assays were executed on a CFX96 System (Biorad, CA, USA). For the relative expression, the results were examined with the ΔΔCt method, considering Gapdh as the internal reference gene.

### 4.7. Statistical Analysis

Statistical analysis was performed by using the GraphPad Prism statistical software release 5.0 for Windows XP. All data were expressed as mean ± SEM. The statistical significance was determined with the two-tailed Student’s *t*-test. Results were considered statistically significant at *p* < 0.05. Experiments were carried out at least three times.

## 5. Conclusions

The in vitro Winnie gut organoid model mimics the in vivo disease setting demonstrating characteristic inflammatory gene regulation of TNF-α, Slpi and Lcn-2. Quercetin regulates epithelial inflammatory genes involved in both the LPS-induced organoid and the Winnie organoid models of inflammation downregulating Lcn-2, TNF-α and C/EBP-β and upregulating Hmox1 and Fpn1.

Winnie gut organoids show a similar morphology to WT-organoids and are a useful model to study gut epithelial inflammatory responses. 

## Abbreviations

IBD	Inflammatory bowel disease
CD	Crohn’s disease
UC	Ulcerative colitis
2-D	Two-dimensional
3-D	Three-dimensional
ESCs	Embryonic stem cells
iPSCs	Induced pluripotent stem cells
BMDCs	Bone marrow dendritic cells
Slpi	Secretory leukoprotease inhibitor
WT	Wild-type
Hmox1	Heme oxygenase 1
Fpn1	Ferroportin-1
C/EBP-β	CCAAT/enhancer binding protein β
Lcn-2	Lipocalin2
DPBS	Dulbecco’s phosphate buffered saline
GCDR	Gentle cell dissociation reagent
